# Air pollution impairs recovery and tissue remodeling in a murine model of acute lung injury

**DOI:** 10.1038/s41598-020-72130-3

**Published:** 2020-09-17

**Authors:** Natália de Souza Xavier Costa, Gabriel Ribeiro Júnior, Adair Aparecida dos Santos Alemany, Luciano Belotti, Alexandre Santos Schalch, Marcela Frota Cavalcante, Susan Ribeiro, Mariana Matera Veras, Esper Georges Kallás, Paulo Hilário Nascimento Saldiva, Marisa Dolhnikoff, Luiz Fernando Ferraz da Silva

**Affiliations:** 1 Laboratório de Poluição Atmosférica Experimental (LIM05), Departamento de Patologia, Faculdade de Medicina da Universidade de São Paulo, Avenida Dr. Arnaldo, 455, São Paulo, São Paulo 01246-903 Brazil; 2 Departamento de Análises Clínicas E Toxicológicas, Faculdade de Ciências Farmacêuticas da, Universidade de São Paulo, São Paulo, São Paulo Brazil; 3 Laboratório de Imunologia Clínica E Alergia (LIM60), Faculdade de Medicina da Universidade de São Paulo, São Paulo, São Paulo Brazil; 4 Department of Pathology, Case Western Reserve University, Cleveland, OH USA

**Keywords:** Respiration, Animal disease models, Acute inflammation

## Abstract

Evidence regarding the impact of air pollution on acute respiratory distress syndrome (ARDS) is limited, and most studies focus on ARDS onset. Our study aimed to evaluate whether exposure to fine particulate matter interferes with lung recovery and remodeling in a murine model of acute lung injury. Forty-eight mice received nebulized LPS or the vehicle (controls). Blood, BALF, lungs and spleen were collected after 5 weeks of exposure to either PM_2.5_ (PM and LPS + PM group) or filtered air (control and LPS5w groups). Inflammatory cells and cytokines were assessed in the blood, BALF, lungs and spleen. Stereological analyses and remodeling assessments were performed by histology. The LPS + PM group showed increased BALF leukocytes, characterized by increased macrophages, increased IL-1β and IL-6 levels, anemia and thrombocytopenia. Moreover, we also observed septal thickening, decreased alveolar air space total volume and, septa surface density. Finally, regarding tissue remodeling, we observed elastosis of the lung parenchyma, and unlike in the LPS5w group, we did not observe fibrosis in the LPS + PM group. In conclusion, the delayed inflammation resolution due to subchronic exposure to PM_2.5_ could be influenced by low systemic and local lymphocyte counts, which lead to impaired lung injury recovery and tissue remodeling.

## Introduction

Exposure to air pollution annually causes 7 million premature deaths from conditions such as cancer, stroke, heart and lung diseases^1^. Moreover, air pollution exposure has also been linked to increased morbidity in individuals with preexisting respiratory diseases^2^. Particulate matter (PM) with an aerodynamic diameter less than or equal to 2.5 µm (fine particulate matter, PM_2.5_) has been shown to induce acute exacerbation and aggravation of respiratory diseases^3^. In addition, PM_2.5_ can penetrate deeply into the lung and irritate and injure the alveolar walls, leading to impaired lung function and finally reaching the circulation. The pathogenicity of PM_2.5_ is determined by its composition, origin, solubility and ability to produce reactive oxygen^4^.

Some experimental studies have addressed the effects of PM on acute lung injury in animal models. Several experimental studies modeling lipopolysaccharide (LPS)-induced acute lung injury exposed animals to different types of PM_2.5_ and observed thickened alveolar walls, neutrophil recruitment, macrophage activation, alveolar edema and hemorrhage, increased oxidative stress and expression of pro-inflammatory cytokines^5–9^.

In humans, Reilly et al.^10^ observed that long-term exposure to low to moderate levels of air pollution is associated with an increased risk of developing acute respiratory distress syndrome (ARDS) after severe trauma. Similarly, Lin et al.^11^ examined the short-term association between air pollution and ARDS. They observed a concentration–response relationship between the mass concentration of different sizes of PM and ARDS morbidity, without clear evidence of threshold concentrations below which PM pollution had no effects.

Although information regarding the effects of air pollution on acute lung injury (ALI)/ARDS onset is available, limited evidence regarding the impact of air pollution on ARDS course and particularly on the late stage of the syndrome exists. We hypothesize that exposure to air pollution after the onset of ALI/ARDS may alter the regular course of the disease. Therefore, this study aimed to evaluate whether the exposure to PM_2.5_ interferes with lung recovery and remodeling in a murine model of acute lung injury.

## Results

### Exposure and PM_2.5_ elementary composition characterization

The concentration of PM_2.5_ is susceptible to fluctuations; therefore, the time of exposure was adjusted accordingly to achieve our target dose. The mean PM_2.5_ dose was 1,211.68 µg m^−3^ and the associated 24-h mean PM_2.5_ exposure was 50.4 µg m^−3^. The air pollution at the exposure site was previously characterized as emission from predominantly vehicular sources^12,13^. The concentration of PM_2.5_ collected in the filters, as well as its black carbon (BC) and metal trace content, was previously published elsewhere by Lopes et al.^14^. The polycyclic aromatic hydrocarbon content of PM_2.5_ was also previously published by Yoshizaki et al.^15^. Endotoxin content was quantified on PM_2.5_ extracted from one of the collected filters. This sample had a concentration of 0.975 toxin units per milligram of PM_2.5_ (EU/mg).

### Body weight

We did not observe weight differences among the groups at the beginning or at the end of the exposure protocol. The body weight gain among the groups also showed no statistical difference. The mean and standard deviation values are presented in the Supplementary Table S1.

### Histopathological characteristics of the lungs

**Figure 1:**
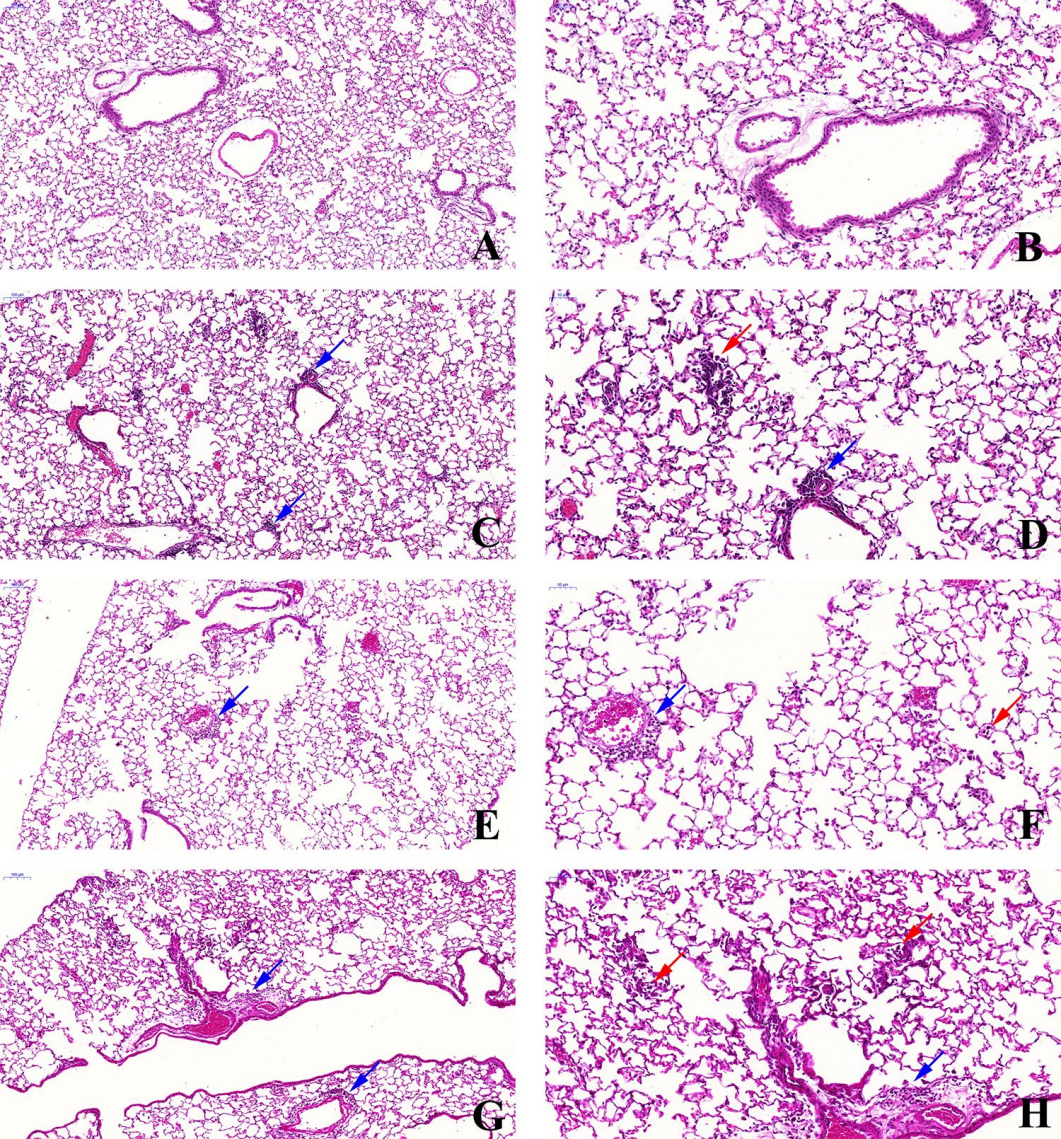
Representative photomicrographs of lung tissue (H & E staining). (**A**) (10 × scale bar 100 μm) and (**B**) (20 ×, scale bar 50 μm)—Control group: thin alveolar septa and no significant inflammation. (**C**) (10 ×, scale bar ×100 μm) and (**D**) (20 ×, scale bar 50 μm)—PM group: perivascular and peribronchial inflammatory infiltration and mild inflammation in the alveolar septa. (**E**) (10 ×, scale bar 100 μm) and (**F**) (20 ×, scale bar 50 μm)—LPS5w group: perivascular and peribronchial inflammatory infiltration and mild inflammation in the alveolar septa, predominantly composed of mononuclear cells. (**G**) (10 ×, scale bar 100 μm) and (**H**) (20 ×, scale bar 50 μm)—LPS + PM group: inflammatory infiltration along the bronchovascular bundle and with moderate presence of inflammatory cells in the alveolar septa, predominantly mononuclear cells. In addition, the lung tissue of the LPS + PM group showed enlarged alveoli with irregular distribution. Blue arrows = infiltration of inflammatory cells in the peribronchial and perivascular areas. Red arrows = interalveolar inflammatory cells.

Macroscopically, the lungs of all groups appeared normal at the time of euthanasia. Microscopically, the lungs of the control group animals exhibited a healthy aspect with no signs of inflammation (Fig. 1A,B). The PM group showed the presence of inflammatory cells in the peribronchial space and mild infiltration in the alveolar septa (Fig. 1C,D). The LPS5w group had perivascular and peribronchial inflammatory infiltration and mild inflammation in the alveolar septa, predominantly composed of mononuclear cells (Fig. 1E,F). The LPS + PM group showed pulmonary tissue with inflammatory infiltration along the bronchovascular bundle and with a moderate presence of inflammatory cells in the alveolar septa, predominantly mononuclear cells. The lung parenchyma of the LPS + PM group also showed enlarged alveoli with irregular distribution (Fig. 1G,H).

Semi-quantitative analysis showed a significant increase in the peribronchial inflammation score in the LPS + PM (*p* = 0.016) and LPS5w (*p* = 0.016) groups compared to the control group, as a result of the LPS nebulization (*p* = 0.001). In addition, we observed a significant increase of the inflammatory cells in the alveolar septa in the LPS5w group compared to the control (*p* = 0.01), PM (*p* = 0.01) and LPS + PM (*p* = 0.01) groups, influenced by the LPS nebulization (*p* = 0.021), PM exposure (*p* = 0.021) and by the interaction between them (*p* = 0.021). The mean and standard deviation values are presented in Table 1.

**Table 1:** Peribronchial and alveolar septa inflammation score and inflammatory cell counts.

	Control	PM	LPS5w	LPS + PM	LPS nebulization	PM2.5 exposure	Interaction
Peribronchial inflammation score	0.25 ± 0.4	0.63 ± 0.5	1.25 ± 0.7^a^	1.25 ± 0.7^a^	*p* = 0.001	n/s	n/s
Alveolar septa inflammation score	0.3 ± 0.5^b^	0.37 ± 0.5^b^	1.25 ± 0.5	0.3 ± 0.5^b^	*p* = 0.021	*p* = 0.021	*p* = 0.021
**Leukocytes**							
Blood (10^3^ cells/mm^3^)	2.61 ± 0.35	4.31 ± 1.58^a,c^	5.44 ± 0.97^a,c^	2.71 ± 0.91	n/s	n/s	*p* ≤ 0.0001
BALF (10^4^ cells/ml)	14.96 ± 3.58^b,c^	21.38 ± 10.67^c^	31.2 ± 10.27^c^	53.7 ± 14.37	*p* ≤ 0.0001	*p* = 0.001	*p* = 0.042
**Neutrophils**							
Blood (10^3^ cells/mm^3^)	0.68 ± 0.18	1.36 ± 0.6^a,c^	1.18 ± 0.22^a,c^	0.56 ± 0.2	n/s	n/s	*p* ≤ 0.0001
BALF (10^3^ cells/ml)	3.23 ± 3.7	9.18 ± 11.7	4.73 ± 3.79	17.68 ± 19.6	n/s	*p* = 0.031	n/s
MPO + cells (10^−5^ cells/µm^2^)	0.67 ± 0.63	0.47 ± 0.31	1.01 ± 0.58	1.14 ± 0.44	n/s	n/s	n/s
**Lymphocytes**							
Blood (10^3^ cells/mm^3^)	1.78 ± 0.2^b^	2.79 ± 1.01^b^	4.12 ± 0.81	1.99 ± 0.66^b^	*p* = 0.008	*p* = 0.048	*p* ≤ 0.0001
BALF (10^3^ cells/ml)	4.76 ± 2.34^b^	3.13 ± 2.2^b^	19.0 ± 14.9	4.72 ± 2.66	*p* = 0.012	*p* = 0.012	*p* = 0.04

Data are expressed in mean ± standard deviation. n/s, not significant. ^a^*p* < 0.05 compared to the control group. ^b^*p* < 0.05 compared to the LPS5w group. ^c^*p* < 0.05 compared to the LPS + PM group.

### Red blood cell counts

Erythrocytes were decreased in the LPS + PM group compared to the control (*p* = 0.026) and LPS5w (*p* ≤ 0.0001) groups and the PM group showed lower erythrocyte count compared to the LPS5w group (*p* = 0.034). The two-way ANOVA analysis showed that this decrease was influenced by the PM exposure (*p* ≤ 0.0001) and by the interaction between the LPS and PM exposure (*p* = 0.01) Additionally, hemoglobin was decreased in the PM group (*p* = 0.031) and in the LPS + PM group (*p* = 0.009) compared to the LPS5w group, as a result of the PM exposure (*p* = 0.002). The mean corpuscular volume (MCV) and the mean corpuscular hemoglobin (MCH) were increased in the LPS + PM group compared to the control (*p* = 0.015 and *p* = 0.008, respectively), PM (*p* ≤ 0.0001 and *p* = 0.001, respectively) and LPS5w (*p* = 0.008 and *p* = 0.014, respectively) groups. The MCV and MCH increase was influenced by the LPS nebulization (*p* = 0.003 and *p* = 0.004, respectively) and by the interaction between the LPS and PM exposure (*p* = 0.001 and *p* = 0.007, respectively). Among the groups, the mean corpuscular hemoglobin concentration (MCHC) was not different. The hematocrit was decreased in the PM group (*p* = 0.012) and in the LPS + PM group (*p* = 0.003) compared to the LPS5w group, as a result of the PM exposure (*p* = 0.001). The fibrinogen levels of PM and LPS + PM groups were decreased compared to the control (*p* ≤ 0.0001 and *p* ≤ 0.0001, respectively) and LPS5w (*p* ≤ 0.0001 and *p* ≤ 0.0001, respectively) groups, influenced by the PM exposure (*p* ≤ 0.0001). The platelet counts of the PM and LPS + PM groups were also decreased compared to the control (*p* = 0.001 and *p* = 0.036, respectively) and LPS5w groups (*p* ≤ 0.0001 and *p* = 0.003, respectively), as a result of the PM exposure (*p* ≤ 0.0001) and LPS nebulization (*p* = 0.043). These results are summarized in the Supplementary Table S2.

### Inflammatory cell counts

The white blood cell count showed leukocytosis in the PM and LPS 5w groups compared to the control (*p* = 0.018 and *p* ≤ 0.0001) and LPS + PM (*p* = 0.035 and *p* ≤ 0.0001) groups. The groups LPS5w (*p* = 0.017) and PM (*p* ≤ 0.0001) had increased neutrophil counts compared to the LPS + PM group. The neutrophil count in the PM group was higher compared to the control (*p* = 0.005) and the LPS5w group showed an increasing tend compared to the control group (*p* = 0.05). The leukocyte and neutrophil counts were influenced by the interaction between the LPS and PM exposure (*p* ≤ 0.0001, for both variables).

The lymphocyte count in the LPS5w group was increased compared to the control (*p* ≤ 0.0001), PM (*p* = 0.009) and LPS + PM (*p* ≤ 0.0001) groups, as a result of the LPS nebulization (*p* = 0.008), PM exposure (*p* = 0.048) and the interaction between them (*p* ≤ 0.0001). There was no difference in the blood monocyte and eosinophil counts among the groups.

The bronchoalveolar lavage fluid (BALF) cell count exhibited increased leukocytes in the LPS5w group compared to the control (*p* = 0.020) group. Furthermore, unlike the blood cell count, the LPS + PM group showed a more pronounced increase in BALF leukocytes than did the control (*p* ≤ 0.0001), the PM (*p* ≤ 0.0001) and LPS5w (*p* ≤ 0.0001) groups, as a result of the LPS nebulization (*p* ≤ 0.0001), PM exposure (*p* = 0.001) and the interaction between them (*p* = 0.042).

**Figure 2:**
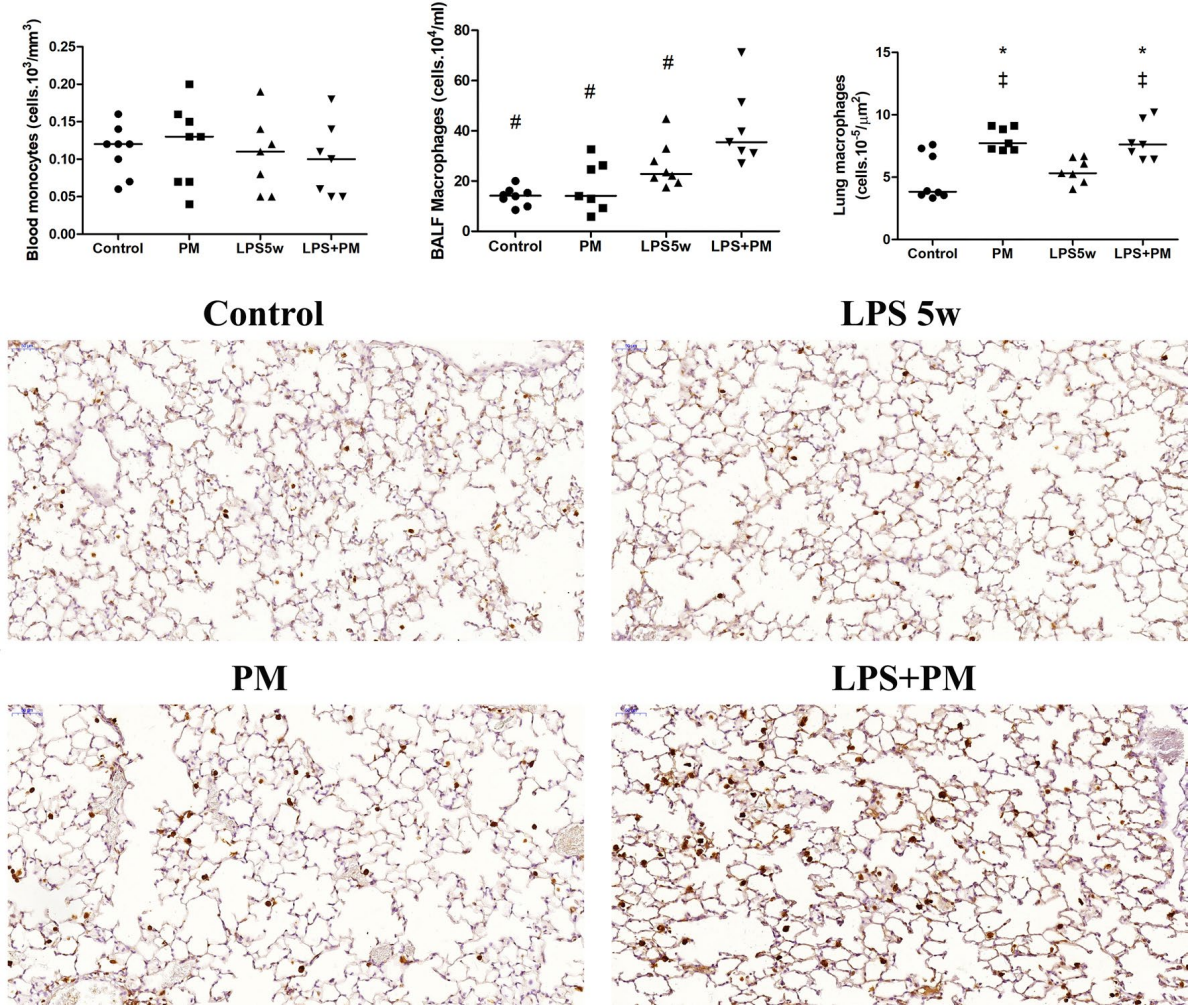
Graphical representation of monocytes in the blood (cells 10^3^/mm^3^), macrophages in the BALF (cells.10^4^/ml) and immunostained macrophages in the lung tissue (cells 10^–5^/µm^2^). Each point represents one animal and bars show median. Representative photomicrographs (20 ×, scale bars 50 μm) of immunostained MAC2-positive macrophages in the lung parenchyma: the LPS + PM group showed increased macrophages, showing the same pattern as the BALF. ^‡^*p* < 0.05 compared to the control group. *p < 0.05 compared to the LPS5w group. #*p* < 0.05 compared to the LPS + PM group. The two-way ANOVA analyses showed that the BALF macrophage count was influenced by the PM exposure and by the interaction between the LPS and PM exposure. The immunostained macrophages were influenced only by the PM exposure.

The increase in the BALF leukocyte count in the LPS5w group was predominantly due to the increased lymphocytes compared to the control (*p* = 0.007), the PM (*p* = 0.004) and LPS + PM groups (*p* = 0.01), influenced by the LPS nebulization (*p* = 0.012), PM exposure (*p* = 0.012) and the interaction between them (*p* = 0.04).Whilst, the increased leukocyte count in the BALF of the LPS + PM group was predominantly due to the increased macrophages compared to the levels in the control (*p* ≤ 0.0001), PM (*p* = 0.001) and LPS5w (*p* = 0.04)) groups (Fig. 2), as a result of the LPS nebulization (*p* ≤ 0.0001) and PM exposure (*p* = 0.017). There was no difference in the BALF neutrophil count among the groups. The inflammatory cell count results are summarized in Table 1.

**Figure 3:**
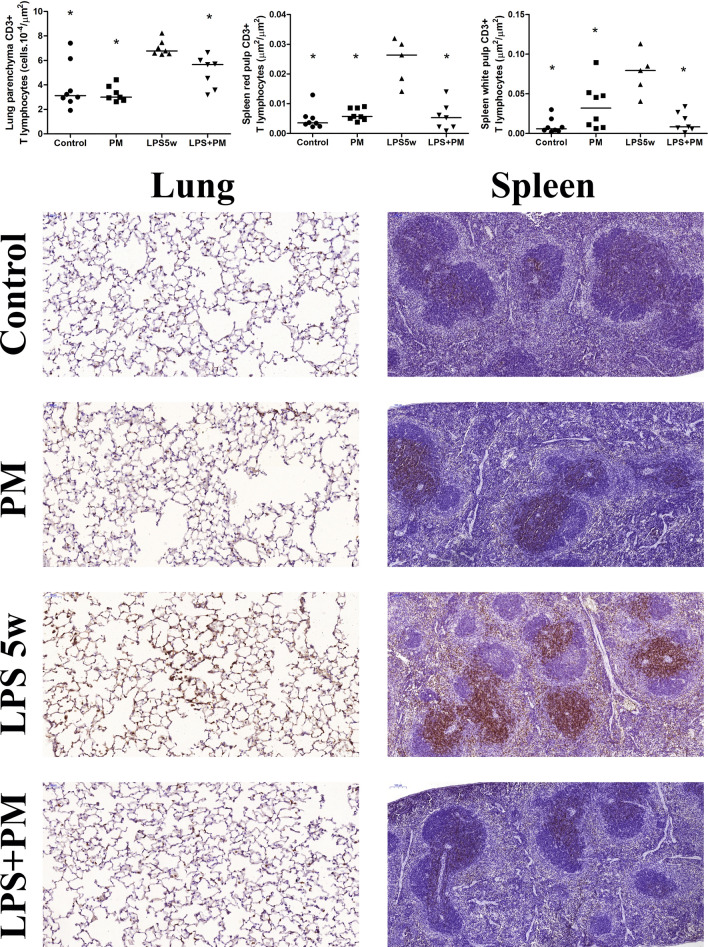
Graphical representation of CD3-positive lymphocytes in the lung parenchyma (cells 10^–4^/µm^2^) and spleen red and white pulp (µm^2^/µm^2^). Each point represents one animal and bars show median. Representative photomicrographs of immunostained CD3 lymphocytes in the lung parenchyma (20 ×, scale bars 50 μm) and spleen (10 ×, scale bars 100 μm): the CD3-positive lymphocyte content of the LPS5w group was increased compared to that of the control, PM and LPS + PM groups. The same pattern was observed for circulating and BALF lymphocytes. *p < 0.05 compared to the LPS5w group. The two-way ANOVA analyses showed that the CD3-positive lymphocyte count in the lung parenchyma was influenced by the LPS nebulization and PM exposure. The spleen CD3-positive lymphocyte counts in the white and red pulp were influenced by the LPS nebulization, PM exposure and their interaction.

The number of MAC 2-positive macrophages in the lung parenchyma was increased in the PM and LPS + PM group compared to the control (*p* = 0.002 and *p* = 0.003) and LPS5w (*p* = 0.014 and *p* = 0.026) groups (Fig. 2), due to the PM exposure (*p* ≤ 0.0001). Myeloperoxidase (MPO)-positive neutrophils in the lung tissue showed no difference among the groups (Table 1). Notably, the circulating and BALF lymphocytes, and the T lymphocytes (CD3-positive) of the lung parenchyma and spleen (white and red pulp) presented the same pattern. The T lymphocytes in the lung parenchyma were increased in the LPS5w group compared to the control (*p* ≤ 0.0001), PM (*p* ≤ 0.0001) and the LPS + PM (*p* = 0.04) groups (Fig. 3), as a result of the LPS nebulization (*p* ≤ 0.0001) and PM exposure (*p* = 0.013). The T lymphocytes in the spleen white and red pulp were increased in the LPS5w group compared to the control (*p* ≤ 0.0001 for both variables), PM (*p* = 0.012 and *p* ≤ 0.0001) and LPS + PM groups (*p* ≤ 0.0001 for both variables) (Fig. 3). The spleen white and red pulp T lymphocytes levels were influenced by the LPS nebulization (*p* = 0.009 and *p* ≤ 0.0001), by the PM exposure (*p* = 0.033 and *p* ≤ 0.0001) and by the interaction between them (*p* ≤ 0.0001 for both variables). The LPS + PM group displayed circulating and CD3-positive T lymphocytes in the lungs and spleen at the same levels as the control group (Fig. 3). The mRNA expression of Foxp3 was increased in the PM group compared to the control group (*p* = 0.032), as a result of the PM exposure (*p* = 0.044).

### Inflammatory cytokines

**Figure 4:**
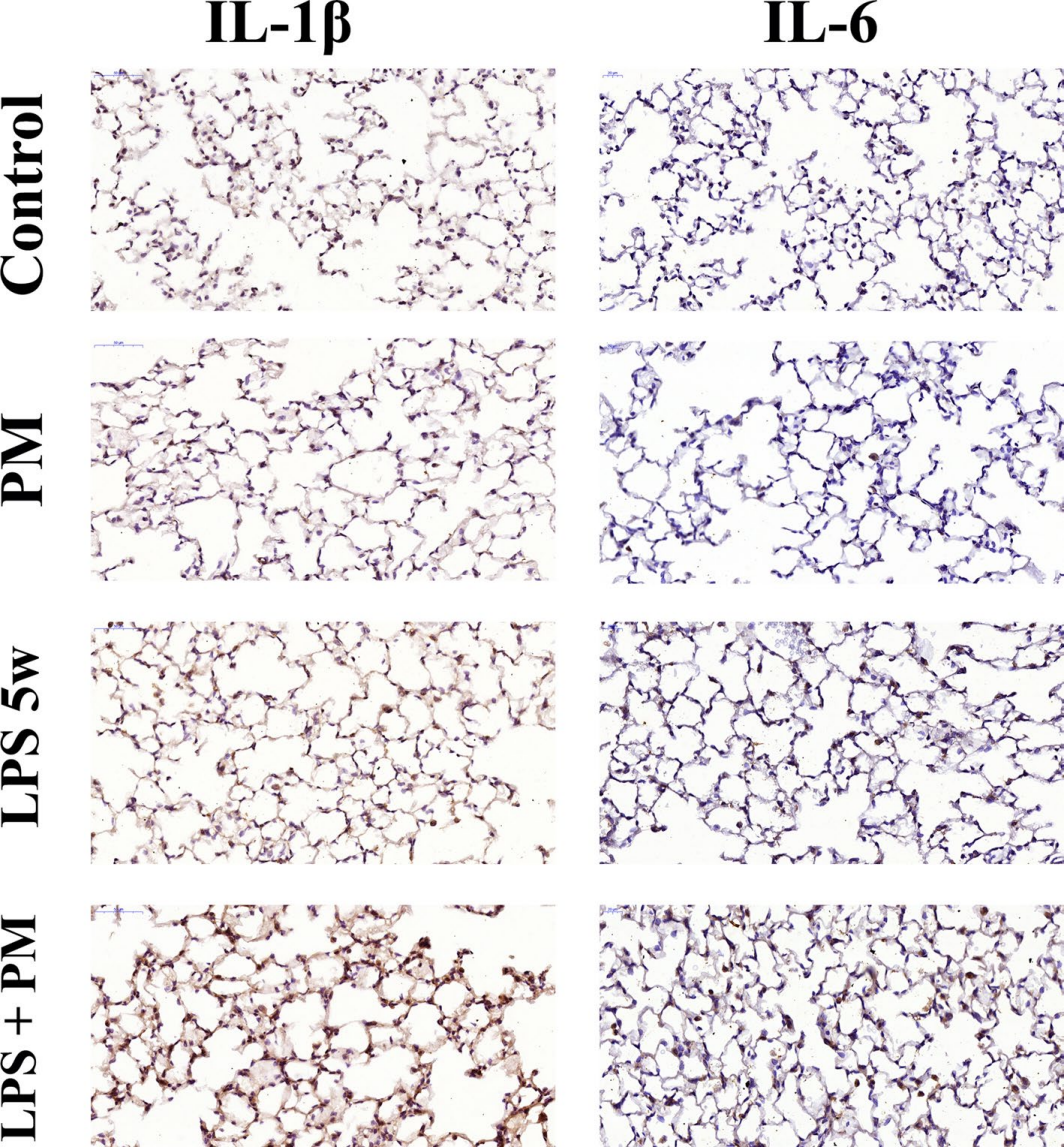
Representative photomicrographs (40 ×, scale bars 20 μm) of the lung parenchyma immunostained with anti-IL-1β and anti-IL-6. Both interleukins were increased in the LPS + PM group; however, mostly epithelial cells are positively stained for IL-1β, while IL-6 positively stained mostly macrophages.

We observed no difference in the IL-1β, IL-6, IL-10 and total TNF levels of blood serum and BALF among the groups. The serum keratinocyte-derived chemokine (KC) levels of the LPS5w group were increased compared to that of the control (*p* = 0.014) and of the PM (*p* = 0.003) groups; however, this difference was not observed in BALF. The serum KC levels was influenced only by the LPS nebulization (*p* ≤ 0.0001). The IL-1β levels in lung tissue were increased in the LPS + PM group compared to the control (*p* = 0.001), PM (*p* ≤ 0.0001) and LPS5w (*p* = 0.013) groups (Fig. 4), as a result of the LPS nebulization (*p* ≤ 0.0001) and of the interaction of the LPS and PM exposure (*p* = 0.007). In addition, the lung tissue of the LPS + PM group showed increased IL-6 levels compared to the lung tissue of the control (*p* ≤ 0.0001), PM (*p* ≤ 0.0001) and LPS5w (*p* ≤ 0.0001) groups (Fig. 4), influenced by the LPS nebulization (*p* ≤ 0.0001), by the PM exposure (*p* = 0.006) and by the interaction between the LPS and PM exposure (*p* ≤ 0.0001). The levels of IL-10 in the lung tissue in the LPS + PM group tended to increased compared to the PM group (*p* = 0.05), as a result of the interaction between the LPS and PM exposure (*p* = 0.015). We observed no difference in the levels of TNF-α in lung tissue among the groups. The inflammatory cytokines results are summarized in the Table 2.

**Table 2:** Inflammatory cytokines.

	Control	PM	LPS5w	LPS + PM	LPS nebulization	PM2.5 exposure	Interaction
**KC**							
Serum (pg/ml)	3.66 ± 1.35^b^	3.19 ± 1.16^b^	7.99 ± 3.82	6.5 ± 1.82	*p* ≤ 0.0001	n/s	n/s
BALF (pg/ml)	1.40 ± 1.72	2.98 ± 3.84	0.83 ± 0.54	0.46 ± 0.24	n/s	n/s	n/s
**IL-1β**							
Serum (pg/ml)	1.91 ± 0.03	> LD	> LD	1.9 ± 0.01	n/s	n/s	n/s
BALF (pg/ml)	1.95 ± 0.12	1.9 ± 0.2	> LD	> LD	n/s	n/s	n/s
Tissue (µm^2^/µm^2^)	0.022 ± 0.014^c^	0.012 ± 0.0034^c^	0.033 ± 0.024^c^	0.080 ± 0.046	*p* ≤ 0.0001	n/s	*p* = 0.007
**IL-6**							
Serum(pg/ml)	1.54 ± 0.19	1.53 ± 0.14	1.52 ± 0.16	1.56 ± 0.37	n/s	n/s	n/s
BALF (pg/ml)	1.4 ± 0.14	1.52 ± 0.19	1.4 ± 0.2	> LD	n/s	n/s	n/s
Tissue (µm^2^/µm^2^)	0.007 ± 0.005^c^	0.001 ± 0.0007^c^	0.008 ± 0.005^c^	0.031 ± 0.011^c^	*p* ≤ 0.0001	*p* = 0.006	*p* ≤ 0.0001
**IL-10**							
Serum (pg/ml)	10.6 ± 1.88	9.92 ± 0.4	10.2 ± 0.58	10.23 ± 0.9	n/s	n/s	n/s
BALF (pg/ml)	10.18 ± 1.22	9.76 ± 0.33	9.77 ± 0.48	9.75 ± 0.35	n/s	n/s	n/s
Tissue (µm^2^/µm^2^)	0.0199 ± 0.0127	0.0045 ± 0.056	0.0077 ± 0.0038	0.048 ± 0.056	n/s	n/s	*p* = 0.015
**TNF**							
Serum (pg/ml)	3.61 ± 0.66	4.12 ± 1.1	3.85 ± 1.08	3.87 ± 0.75	n/s	n/s	n/s
BALF (pg/ml)	2.98 ± 0.26	3.19 ± 0.55	> LD	2.82 ± 0.04	n/s	n/s	n/s
Tissue (µm^2^/µm^2^)	0.055 ± 0.038	0.049 ± 0.029	0.078 ± 0.053	0.056 ± 0.029	n/s	n/s	n/s

Data are expressed in mean ± standard deviation. > LD, below the limit of detection; n/s, not significant. ^b^*p* < 0.05 compared to the LPS5w group. ^c^*p* < 0.05 compared to the LPS + PM group.

### Stereological analysis

The ANOVA statistical test showed a difference among the groups in the total lung volume (*p* = 0.04) and the lung volume per body weight ratio (*p* = 0.029); however, it did not show statistical difference in the post-hoc analysis.

The parenchyma volume density was not statistically different among the groups. The septa volume density was increased in the LPS + PM group compared to the control (*p* = 0.04) group, influenced by the LPS nebulization (*p* = 0.031). The septa total volume was increased in the LPS5w group compared to the control group (*p* = 0.024) and the ANOVA two-way analysis showed an interaction between the LPS and PM exposure (*p* = 0.002) The volume density of alveolar air space was decreased in the LPS + PM group compared to LPS5w (*p* = 0.049) groups, as a result of the PM exposure (*p* = 0.01). The total volume of alveolar air space was increased in the LPS5w group compared to the LPS + PM (*p* = 0.02) group, influenced by the interaction between the LPS and PM exposure (*p* = 0.004).

The total volume and the volume density of the non-parenchyma structures were not different among the groups.

The septa surface density was decreased in the LPS + PM group compared to the control group (*p* = 0.009), influenced by the PM exposure (*p* = 0.004); however, the total surface area had no difference among the groups. Furthermore, was decreased in the LPS + PM group compared to the LPS5w (*p* = 0.015) group.

We observed an alveolar septa thickening in the LPS + PM group compared to the control group (*p* = 0.005) and a tendency compared to the PM group (*p* = 0.05). The alveolar septa thickness was influenced by the LPS nebulization (*p* = 0.029) and by the PM exposure (*p* = 0.005). The lung stereology parameters are summarized in Table 3.

**Table 3:** Stereological measures of the lungs.

Parameter	Control	PM	LPS5w	LPS + PM	LPS nebulization	PM_2.5_ exposure	Interaction
Lung volume (mm^3^)	439.04 ± 85.47	535.08 ± 151.66	540.96 ± 48.87	412.16 ± 98.63	n/s	n/s	*p* = 0.005
Lung volume per body weight ratio (mm^3^/g)	18.47 ± 3.26	20.08 ± 5.24	22.51 ± 2.28	17.13 ± 3.81	n/s	n/s	*p* = 0.017
Vv parenchyma (%)	78.3 ± 5.4	79.6 ± 3.5	83.2 ± 4.7	78.1 ± 8.8	n/s	n/s	n/s
Vv septa (%)	29.82 ± 4.14	33.36 ± 2.45	34.16 ± 2.47	34.9 ± 4.82^a^	*p* = 0.031	n/s	n/s
Vv alveolar airspace (%)	48.6 ± 2.13	46.24 ± 4.35	49.05 ± 4.61^b^	43.14 ± 5.05	n/s	*p* = 0.010	n/s
Vv non-parenchyma (%)	21.6 ± 5.4	20.3 ± 3.5	16.7 ± 4.7	21.8 ± 8.8	n/s	n/s	n/s
Vt parenchyma (mm^3^)	344.08 ± 68.49	426.95 ± 124.57	450.82 ± 53.98^b^	320.18 ± 79.42	n/s	n/s	*p* = 0.002
Vt septa (mm^3^)	130.22 ± 26.75	176.85 ± 44.00	184.73 ± 21.05^a^	143.86 ± 42.11	n/s	n/s	*p* = 0.002
Vt alveolar airspace (mm^3^)	213.85 ± 45.28	250.10 ± 82.51	266.09 ± 40.56^b^	176.32 ± 39.14	n/s	n/s	*p* = 0.004
Vt non-parenchyma (mm^3^)	94.95 ± 32.3	108.12 ± 32.82	90.13 ± 23.2	91.97 ± 45.09	n/s	n/s	n/s
Sv septa (mm^−1^)	243.07 ± 28.84	213.01 ± 28.29	223.16 ± 6.48	201.44 ± 21.06^a^	n/s	*p* = 0.004	n/s
St septa (10^3^ mm^2^)	107.91 ± 31.39	114.69 ± 37.5	120.61 ± 10.12	83.21 ± 21.8	n/s	n/s	n/s
Septal thickness (µm)	2.50 ± 0.5	3.20 ± 0.6^a^	3.06 ± 0.2	3.5 ± 0.5^a^	*p* = 0.029	*p* = 0.005	n/s

Data are expressed in mean ± standard deviation. *Vv*, volume density; Vt, total volume; Sv, Estimated surface area per unit of volume; St, total surface area; n/s, not statistically significant. ^a^*p* < 0.05 compared to the control group. ^b^*p* < 0.05 compared to the LPS + PM group.

The spleen total volume was higher in the LPS5w group than in the control (*p* = 0.002), PM (*p* ≤ 0.0001) and LPS + PM (*p* ≤ 0.0001) groups. We also observed a significant decrease in the LPS + PM group compared to the control (*p* = 0.009) and PM (*p* = 0.023) groups. The spleen weight per body weight ratio was increased in the LPS5w group compared to the control (*p* = 0.001), PM (*p* ≤ 0.0001) and LPS + PM (*p* ≤ 0.0001) groups. Moreover, this ratio was decreased in the LPS + PM group compared to the control group (*p* = 0.003). Both, the spleen volume and the spleen weight per body weight ratio, were influenced by the PM exposure (*p* ≤ 0.0001, for both variables) and by the interaction between the LPS and PM exposure (*p* ≤ 0.0001, for both variables). The total volume of the red pulp was increased in the LPS5w group compared to the control (*p* = 0.001), PM (*p* ≤ 0.0001) and LPS + PM (*p* ≤ 0.0001) groups. In addition, the total volume of the red pulp was decreased in the LPS + PM group compared to the control (*p* = 0.006) and PM (*p* = 0.023) groups, as a result of the PM exposure (*p* ≤ 0.0001) and of the interaction between the LPS and PM exposure (*p* ≤ 0.0001) The total volume of the white pulp tend to increase in the LPS5w group compared to the LPS + PM (*p* = 0.05) group. The spleen stereological measures are summarized in Table 4.

**Table 4:** Stereological measures of the spleen.

Parameter	Control	PM	LPS5w	LPS + PM	LPS nebulization	PM_2.5_ exposure	Interaction
Spleen Volume (mm^3^)	53.36 ± 7.07^a,b^	51.65 ± 5.87^a,b^	68.27 ± 7.16	40.29 ± 7.52 ^a^	n/s	*p* ≤ 0.0001	*p* ≤ 0.0001
Spleen weight per body weight ratio (mm^3^/g)	0.0023 ± 0.0003^a,b^	0.0020 ± 0.0002^a^	0.0029 ± 0.0003	0.0017 ± 0.0002^a^	n/s	*p* ≤ 0.0001	*p* ≤ 0.0001
Vv white pulp (%)	28.9 ± 4.3	30.4 ± 6.5	26.5 ± 4.9	32.3 ± 4.0	n/s	n/s	n/s
Vv red pulp (%)	66.7 ± 3.4	65.2 ± 5.6	69.9 ± 4.1	62.2 ± 3.3^a^	n/s	*p* = 0.006	n/s
Vt white pulp (mm^3^)	16.5 ± 3.1	16.8 ± 4.9	19.5 ± 3.6	13.7 ± 2.5	n/s	n/s	*p* = 0.044
Vt red pulp (mm^3^)	37.5 ± 5.7^a,b^	35.6 ± 4.1^a,b^	50.6 ± 6.6	26.7 ± 5.5^a^	n/s	*p* ≤ 0.0001	*p* ≤ 0.0001

Data are expressed in mean ± standard deviation. Vv, volume density; Vt, total volume; n/s, not statistically significant. ^a^*p* < 0.05 compared to the LPS5w group. ^b^*p* < 0.05 compared to the LPS + PM group.

### Lung tissue remodeling

**Figure 5:**
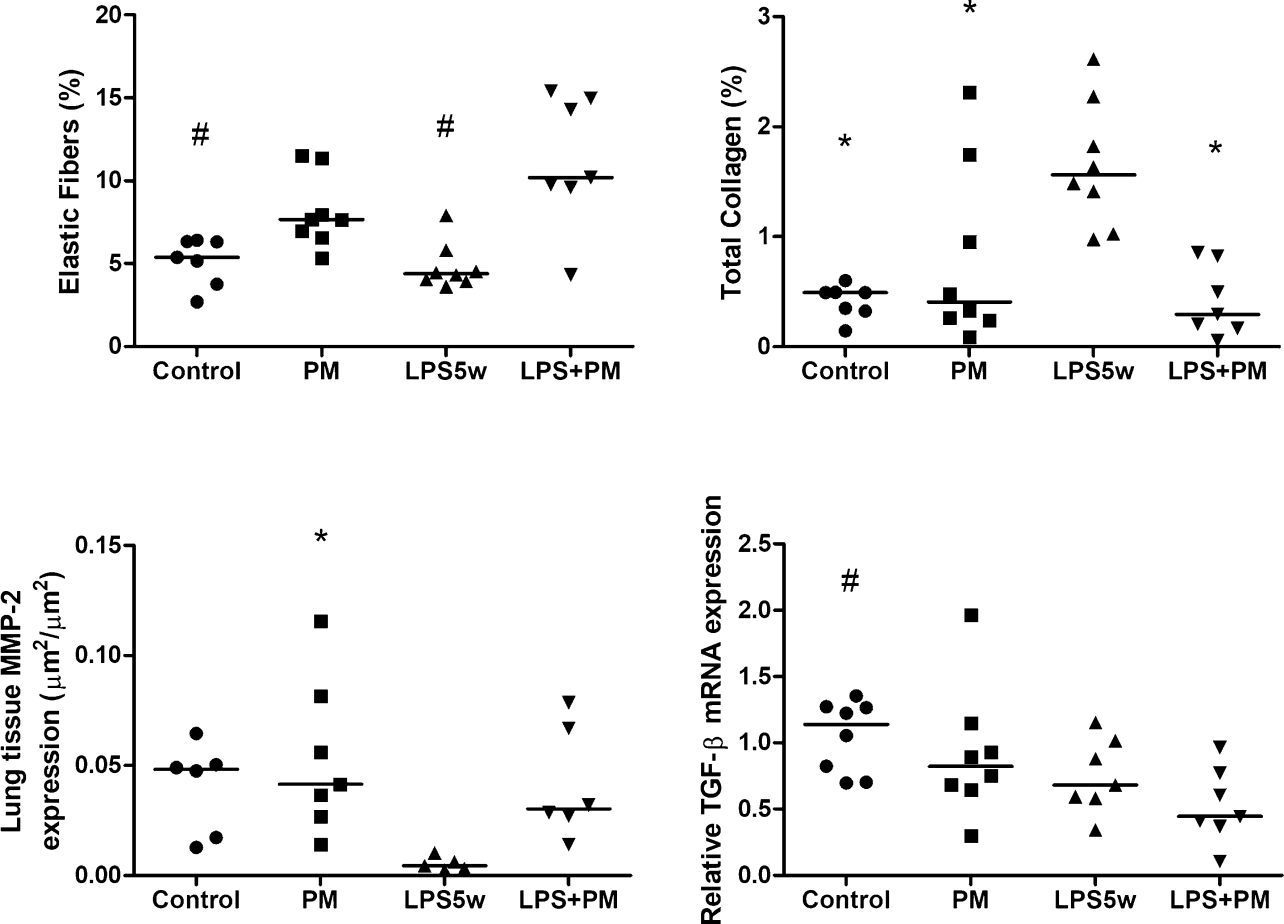
Graphical representation of the elastic fiber content in the lung parenchyma (%), the total collagen content in the lung parenchyma (%), the MMP-2 immunostained area (µm^2^/µm^2^) and relative TGF-β mRNA expression of the lung tissue. Each point represents one animal and bars show median. *p < 0.05 compared to the LPS5w group. #*p* < 0.05 compared to the LPS + PM group. The two-way ANOVA analyses showed that the percentage of elastic fibers was influenced only by the PM exposure. The percentage of the collagen content was influenced by the LPS nebulization and by the interaction between the LPS and PM exposure. The MMP-2 expression in the lung tissue was influenced by the LPS nebulization and PM exposure. The mRNA expression of the TGF-β was influenced only by the LPS nebulization.

The elastic fiber content of the lung parenchyma was significantly increased in the LPS + PM group compared to the control (*p* ≤ 0.0001) and LPS5w (*p* ≤ 0.0001) groups, as a result of the PM exposure (*p* ≤ 0.0001). The total collagen content of the lung parenchyma was increased in the LPS5w group compared to the control (*p* ≤ 0.0001), the PM (*p* = 0.02) and LPS + PM (*p* = 0.002) groups. The collagen content was influenced by the LPS nebulization (*p* = 0.024) and by the interaction between the LPS and PM exposure (*p* ≤ 0.0001). Matrix metalloproteinase-2 (MMP-2) protein expression was decreased in the LPS5w group compared to the PM group (*p* = 0.02), as a consequence of the PM exposure (*p* = 0.029) and LPS nebulization (*p* = 0.035). The gene expression of TGF-β was decreased in the LPS + PM group compared to the control group (*p* = 0.034), influenced by the LPS nebulization (*p* = 0.12) (Fig. 5).

## Discussion

Air pollution is a pro-inflammatory agent that has the potential to induce responses in multiple organs, especially in the respiratory and cardiovascular systems^16^. Our results suggest that exposure to PM_2.5_ after LPS-induced acute lung injury may delay the recovery of the lung tissue. In a 5-week period, we observed anemia, thrombocytopenia, and lung inflammation in the LPS + PM group. The evaluation of inflammatory cell recruitment into the bronchoalveolar space showed leukocytosis, characterized mainly by increased macrophages. We observed increased levels of IL-1β and IL-6 in the lung parenchyma. Moreover, we also observed septal thickening, decreased total volume of alveolar air space and decreased septa surface density. Finally, regarding tissue remodeling, we observed elastosis of the lung parenchyma, and unlike in the LPS5w group, we did not observe fibrosis in the LPS + PM group.

The animals were exposed to an equivalent mean 24-h PM_2.5_ concentration of 50.4 µg m^−3^, which is twofold higher than the 24-h mean level of 25 µg m^−3^ recommended by the World Health Organization^17^. The air quality standards of São Paulo State (Brazil) recommend that the 24-h mean PM_2.5_ concentration should not exceed 60 µg m^−3^, and the air quality index is calculated according to the levels of several atmospheric pollutants, including PM_2.5_. Although the air quality of a station is based on the levels of all monitored pollutants, the pollutant with the highest index (the worst case) determines its classification. The classification of 50.4 µg m^−3^ of PM_2.5_ is in the range between moderate and bad air quality in São Paulo City, and from 2013 to 2017, the annual percentage distribution of days that had moderate air quality or worse in the monitoring stations ranged from 15 to 28.6%^18^.

Some of the effects observed could be related to the air pollution itself, without the LPS interference, such as the anemia and elastosis displayed by the PM and LPS + PM groups. Previous studies have observed higher prevalence of anemia, lower hemoglobin levels and decreased red cell count associated to exposure to higher PM_2.5_ levels in an older population^19^ and in children^20^. Regarding the elastosis, another study from our group showed that the exposure to the São Paulo City air pollution promoted elastic fibers’ deposition in almost all sized arterioles from both ventricles^21^; however, the mechanism by which the particulate matter could promote elastosis remains to be elucidated.

Previous studies showed that air pollution and diesel PM could worsen LPS-induced acute lung injury, leading to an increase in neutrophil recruitment, interstitial edema, alveolar hemorrhage and proinflammatory cytokine expression^7–9^. Additionally, the worsening of lung injury was PM dose-dependent^22^, and the smaller the particle is, the more evident the lung injury^23^. However, these studies only evaluated acute lung injury in an acute PM exposure scenario (up to 24 h). To the best of our knowledge, this is the first study to evaluate the subchronic effects of PM_2.5_ exposure on recovery from acute lung injury.

The LPS + PM group showed persistent inflammation characterized by BALF leukocytosis, increased IL-1β and IL-6 levels in the lung parenchyma, decreased alveolar air space volume, septal thickening and decreased septa surface density. Most of the stereological findings may be explained by the observed active inflammatory process. The septal thickening could be, at least partially, related to edema. Regardless of leukocytosis, BALF differential analysis in the LPS + PM group showed that the total number of lymphocytes did not increase as in the LPS5w group and remained at the same levels as the control group. Furthermore, circulating and BALF lymphocytes and T lymphocytes in the lung tissue and spleen were lower in the LPS + PM group than in the LPS5w group, and the levels in the LPS + PM group were similar to those in the control group. The delayed inflammation resolution could be, at least partially, explained by this systemic low count of the lymphocytes.

Studies with lymphocyte-deficient Rag-1^-/-^ mice have shown an important role of lymphocytes in the resolution of inflammation. In the study conducted by D’Alessio et al.^24^, Rag-1^−/−^ and wild-type mice exhibited a similar extension of LPS-induced lung injury; however, Rag-1^-/-^ mice showed impaired lesion recovery, suggesting that lymphocytes are required for injury resolution. Curiously, these animals showed a sustained increase in BALF macrophages at 4 and 10 days after LPS instillation, and yet, they had impaired injury recovery.

Alveolar macrophages are the major innate immune effector in the cellular response in the alveolar space and are critically important for the removal of the PM from the lung^25^. Macrophages have functional and phenotypic plasticity that becomes apparent during inflammation resolution^26^. Upon apoptotic cell efferocytosis, macrophages, especially M2, turn off the production of proinflammatory cytokines and lipid mediators and start an anti‐inflammatory transcriptional program characterized by the release of IL-10 and TGF‐β^27^, both of which are not increased in the LPS + PM group, although macrophages are increased. A study conducted by Renwick et al.^28^ showed that ultrafine BC particles cause inflammation, induce epithelial damage, significantly impair alveolar macrophage phagocytosis and enhance the sensitivity of alveolar macrophages to C5a chemotaxis, suggesting that exposed macrophages could be more likely to be retained in the lungs. In addition, exposure to PM could not only impair phagocytosis but also pathogen opsonization, contributing to impaired bacterial clearance^25^. Becker et al.^29^ showed that alveolar macrophages exposed to fine PM exhibit reduced phagocytic activity and decreased expression of the receptor CD11b involved in phagocytosis of opsonized microorganisms, yeast wall glucans and LPS.

In addition, previous studies have shown that PM is able to induce a response of alveolar macrophages through toll-like receptors (TLRs) 2 and 4, which recognize the microbial components in the PM, such as endotoxin^30–32^. Our endotoxin analysis of PM_2.5_ confirmed that there are endotoxins in the PM to which our animals were exposed. Nomura et al.^33^ showed that macrophages exposed to low levels of LPS show reduced responses to second stimulation with LPS by downregulating TLR4.

The LPS5w group showed characteristics consistent with the usual course of ARDS. In humans, the rate of ARDS recovery and the underlying conditions that could lead to lung fibrosis are highly variable and controversial^34^; increased lung collagen content is frequently observed in ARDS patients who survive two weeks or more^35,36^. In the LPS5w group, we observed marked fibrosis (4.5-fold compared to the control group); however, in the LPS + PM group, this level of collagen deposition was not observed. The low MMP-2 levels in the LPS5w group may have reduced denatured collagen and collagen fragment degradation^37^ hence contributing to fibrosis.

TGF-β can increase the transcriptional activation of collagen genes, in particular collagen 1, collagen 5 and collagen 6; furthermore, the overproduction or potentiation of pro-fibrotic TGF-β effects leads to an aberrant wound healing response during the early stages of the fibrotic process^38^. The decrease in TGF-β expression in the LPS + PM group may have contributed to the decline in collagen deposition in the lung tissue. In addition, TGF-β activation can be proteolytically mediated by MMP-9 and MMP-2^39^ and we also observed no difference in MMP-2 protein expression in the LPS + PM group.

ARDS-associated lung fibrosis is linked to poor outcomes^40^ and ARDS survivors frequently have sequelae that seriously affect their quality of life^41^. Fibrosis occurs due to the transition of M1 macrophages to the anti-inflammatory profibrotic M2 phenotype and the resulting imbalance of macrophage-regulated inflammatory signaling^42^. Moreover, the persistence of M2 macrophages at the injury sites is a hallmark of the development of fibrosis through TGF-β and arginase 1 pathways^43^. Although we did not directly evaluate macrophage activity, our data suggest that the macrophage response and lymphocyte activation were impaired. However, our data do not allow us to affirm that exposure to PM_2.5_ permanently impairs the inflammation resolution or the collagen deposition in the lung tissue, as we only examined the exposed animals for the limited period of 5 weeks.

The fact that there are no animal models that capture the multifactorial nature of ARDS is probably the most important limitation of our study. Animal models offer controlled conditions to test and validate hypotheses without interfering variables and confounding factors, generating reproducible results. Hence, the ALI model that we used to assess the effects of PM_2.5_ is clearly useful, but far from a perfect model for human conditions. Furthermore, all the employed methodology was selected taking into account the availability of our materials and resources. Among the strengths of our study, we can highlight the exposure to PM_2.5_, that mimics the real-world exposure and that all the analyses were performed only the lung parenchyma, considering that the hallmark of ARDS/ALI pathophysiology is the diffuse alveolar injury.

In conclusion, the delay in the inflammation resolution following subchronic exposure to PM_2.5_ after ARDS onset was possibly influenced by low systemic and local lymphocyte counts, which lead to impaired lung injury recovery and tissue remodeling.

## Materials and methods

### Animals

All ethics aspects were approved by the University of São Paulo—School of Medicine Institutional Review Board for Ethics on Animal Use (protocol no. 177/10), including best practices on animal manipulation and euthanasia. All animals were treated according to the institutional guidelines for animal welfare, with due consideration to the alleviation of distress and discomfort.

Sixty-four BALB/c male mice (9 weeks old) were obtained from our university’s animal facility and maintained at 22–26 °C and 55–75% humidity under a 12/12 h dark/light cycle with food and water provided ad libitum.

### Study design

This experimental study was designed to investigate the effects of exposure to fine concentrated particulate material (PM_2.5_) on LPS-induced lung injury recovery. The acute lung injury was induced with nebulized LPS (lipopolysaccharides from *Escherichia coli* O111:B4—Sigma-Aldrich) at a concentration of 3 mg/ml as previously described by Costa et al.^44^.

Therefore, the following groups (n = 16 each) were established:

Control—Mice exposed to nebulized saline and exposed to filtered air for 5 weeks.

PM—Mice exposed to nebulized saline and exposed to PM_2.5_ (daily dose: 1,200 µg m^−3^) for 5 weeks.

LPS 5w—Mice exposed to nebulized LPS (dose: 3 mg ml^−1^; 5 ml final volume) and exposed to filtered air for 5 weeks.

LPS + PM—Mice exposed to nebulized LPS (dose: 3 mg ml^−1^; 5 ml final volume) and exposed to PM_2.5_ (daily dose: 1,200 µg m^−3^) for 5 weeks.

All animals were euthanized with intraperitoneal injection of sodium thiopental (200 mg kg^−1^ body weight) 24 h after the last exposure to PM_2.5_ or filtered air. Blood samples were collected from all animals and lung samples from 8 animals per group were frozen. From the remaining animals (n = 8 per group), BALF was collected, and the lungs and spleens were fixed in 4% buffered paraformaldehyde solution.

### PM_2.5_ exposure

Animals were exposed to ambient PM_2.5_ in an ambient particle concentrator developed at the Harvard School of Public Health (HAPC). The ambient particle concentrator is described in details in Sioutas et al.^45^. Briefly, the HAPC system consists of a high-volume impactor that samples particles smaller than 2.5 μm, a series of three virtual impactors and the exposure chambers. The whole process occurs without changing the physical characteristics and chemical composition of the particles. The HAPC is located on the campus of the School of Medicine of São Paulo University, close to a high-traffic road (23°33′18.1′'S 46°40′15.0′'W). Animals were placed in exposure chambers with temperature and humidity controlled, either connected to the concentrate PM_2.5_ stream (PM and LPS + PM groups) or to a clean air supply provided using a high-efficiency particulate air filter (control and LPS 5w groups). The atmospheric pressure inside both exposure chambers were regulated to be identical. During the exposure, the mass concentrations of PM were measured using an airborne particulate monitor (two-wavelength nephelometer, DataRam DR-4000, Thermo Fisher Scientific, Waltham, MA, USA), and our target dose was 1,200 µg m^−3^ for 1 h daily. The exposure took place during the dry season and to ensure the best constancy in the delivered PM_2.5_ dose, the exposure time was proportionally adjusted to the HAPC-derived PM_2.5_ concentration at the start of each exposure, not exceeding 120 min of exposure per day.

PM_2.5_ was collected in polycarbonate filter membranes. The concentrations of metal trace elements (Na, Al, Si, P, S, K, Ca, Ti, V, Fe, Nu, Cu, Zn, and Pb) were assessed by X-ray fluorescence spectrometry and a smoke stain reflectometer was used to determine the black carbon (BC) concentration as previously described by Andrade et al.^12^ and de Miranda et al.^13^. The polycyclic aromatic hydrocarbon content of PM_2.5_ was assessed as previously described by Yoshizaki et al.^15^. The endotoxin content in the PM was quantified as recommended by the ToxinSensor Chromogenic LAL Endotoxin Assay Kit (GenScript, Piscataway, NJ, USA).

### Total blood, serum and BALF analysis

We performed full and differential cell counts in the total blood and BALF samples. Differential cell counts were performed using May-Grünwald-Giemsa stain (300 cells per animal). In addition, in the BALF and blood serum, the inflammatory cytokines IL-1β, IL-6, IL-10, KC and total TNF were quantified by the cytometric bead assay (BD Bioscience, CA, USA) according to the manufacturer’s instructions. In this assay, 1,200 events were acquired by a BD FACSCanto II flow cytometer (BD Biosciences, CA, USA), and we analyzed the data with FCAP Array software (BD Biosciences, CA, USA).

### Stereological analysis and descriptive analysis

Lung stereology was conducted as described in Hsia et al.^46^ using newCAST software (Visiopharm, Hørsholm, Denmark). Briefly, lungs were sampled using a stereological approach, fixed in 4% buffered paraformaldehyde solution and embedded in paraffin. Five-micrometer-thick sections were stained with hematoxylin & eosin (H&E) for lung structure analysis. The total lung volume, volume density and total volume of the lung compartments (septa, alveolar spaces and airways) were estimated by the Cavaliere principle. The density surface, the total surface area of the alveolar septa and the arithmetic mean thickness were also assessed as described in Hsia et al.^46^. The spleens were weighed, and the total volume was calculated according to Weibel^47^: V≈W. The red and white pulp volumes were also assessed by the Cavaliere principle.

Furthermore, in the H&E slides, a semi-quantitative analysis of the inflammation was performed by an experienced pathologist to determine histopathological characteristics using the following scores: grade 0 (absent), 1 (discrete), 2 (mild), 3 (moderate) and 4 (intense)^48,49^.

### Molecular analysis

Foxp3 and TGF-β mRNA were quantified by real-time PCR using specific primers: Foxp3: forward primer 5′-TTCATGCATCAGCTCTCCAC-3′ and reverse primer 5′-CTGGACACCCATTCCAGACT-3′; TGF-β: forward primer 5′-ATACGCCTGAGTGGCTGTCT-3′ and reverse primer 5′-TCTCTGTGGAGCTGAAGCAA-3′. Following the manufacturer’s instructions, total RNA of the frozen lung tissue was extracted with TRIzol (Ambion, Life Technologies, Carlsbad, CA, USA). cDNA synthesis (SuperScript VILO cDNA Synthesis Kit, Invitrogen, Life Technologies, Carlsbad, CA, USA) and real-time PCR (Fast SYBR Green Master Mix, Applied Biosystems, Life Technologies, Carlsbad, CA, USA) were also conducted according to the manufacturer’s protocols. The relative expression of the transcripts was calculated after normalization to the levels of the reference gene of the ribosomal protein L13A (forward primer 5′-AACCTTTGGTCCCCACTTCCCT-3′ and reverse primer 5′-TCCTCAAGACCAACGGACTCCT-3′)^50^.

### Immunohistochemical and extracellular content assessment

Lung tissue sections were immunostained using anti–MPO, anti-CD3 and anti-MAC2 antibodies. Immunostained cells were counted in 20 high power fields (HPFs) and the proportion of cells per area of lung tissue was calculated. Lung tissue was also immunostained using anti-IL-1β, anti-IL-6, anti-IL-10, anti-TNF-α and anti-MMP2 antibodies. The spleen slides were immunostained with anti-CD3. The immunostained CD3 in the spleen, MMP-2 and the inflammatory cytokines in the lung parenchyma were quantified by measuring the proportional area (stained area/lung tissue area) in 15 HPFs per animal using the Image-Pro Plus 4.1 software (Media Cybernetics, Silver Spring, MD, USA). The commercial sources and the standardized dilutions of the antibodies are presented in the Supplementary Table S3.

Lung tissue slides were stained with Sirius Red to assess collagen content, and Weigert’s resorcin-fuchsin with oxidation to assess elastic fibers content. The proportional areas of collagen and elastic fibers were quantified using the same software and strategy used to analyze the inflammatory cytokines in the immunohistochemistry assay.

### Statistical analysis

SPSS 21 software (SPSS Inc/IBM Chicago, USA) was used for the statistical analyses and the GraphPad Prism 7 (GraphPad Software, La Jolla, CA, USA) was used for data visualization. The mean, median, standard error, standard deviation and interquartile range were calculated for each variable and group. The data distribution was assessed by the Kolmogorov–Smirnov normality test. We performed ANOVA or the Kruskal–Wallis test, followed by Tukey or Bonferroni post hoc tests to compare the groups according to the normality of distribution. In addition, we performed a two-way analysis of variance (two-way ANOVA) to verify the effects of the LPS nebulization, PM_2.5_ exposure and their interaction on each variable. Statistical differences were assumed at the 5% significance level.

## Supplementary information

Supplementary information.
