# Supplementary material for: Air pollution impairs recovery and tissue remodeling in a murine model of acute lung injury
Source: Sci Rep. 2020 Sep 17;10:15314. doi: 10.1038/s41598-020-72130-3 (PMC7499199; doi:10.1038/s41598-020-72130-3)
Supplement: Supplementary file 1 — Supplementary information. [file 41598_2020_72130_MOESM1_ESM.pdf]

Air pollution impairs recovery and tissue remodeling in a murine model of acute lung injury

Natália de Souza Xavier Costa[1*], Gabriel Ribeiro Júnior[1], Adair Aparecida dos Santos Alemany[1], Luciano Belotti[1], Alexandre Santos Schalch[1], Marcela Frota Cavalcante[2], Susan Ribeiro[3,4], Mariana Matera Veras[1], Esper Georges Kallás[3], Paulo Hilário Nascimento Saldiva[1], Marisa Dolhnikoff[1], Luiz Fernando Ferraz da Silva[1].

[1] Laboratório de poluição atmosférica experimental (LIM05), Faculdade de Medicina da Universidade de São Paulo, São Paulo, São Paulo, Brazil;

[2] Departamento de Análises Clínicas e Toxicológicas, Faculdade de Ciências Farmacêuticas da Universidade de São Paulo, São Paulo, São Paulo, Brazil;

[3] Laboratório de Imunologia Clínica e Alergia (LIM60), Faculdade de Medicina da Universidade de São Paulo, São Paulo, São Paulo, Brazil;

[4] Department of Pathology, Case Western Reserve University, Cleveland, Ohio, United States of America.

Correspondence: Departamento de Patologia, Faculdade de Medicina da Universidade de São Paulo. Avenida Dr. Arnaldo, 455, São Paulo, São Paulo 01246-903, Brazil. *e-mail: nataliasxcosta@usp.br

Table S1. Body weight (g) at the beginning and at the end of the exposure protocol, body weight gain (g).

	Control	PM	LPS 5w	LPS + PM	LPS nebulization	$PM_{2.5}$ exposure	Interaction
**Initial body weight (g)**	21.8 ± 1.7	22.7 ± 1.4	21.2 ± 1.4	21.2 ± 2.6	n/s	n/s	n/s
**Final body weight (g)**	24.2 ± 1.6	25.4 ± 2	24.2 ± 0.8	25.3 ± 1.8	n/s	n/s	n/s
**Weight gain (g)**	2.4 ± 1.1	2.67 ± 2.2	2.9 ± 1.4	4.2 ± 2	n/s	n/s	n/s

The results are expressed as the means ± standard deviations. n/s = not statistically significant.

Table S2. Red blood cell count, fibrinogen (mg/dL) and platelet (1000/mm³) quantification.

	Control	PM	LPS 5w	LPS + PM	LPS nebulization	PM 2.5 exposure	Interaction
**Erythrocytes (millions/mm³)**	9.42±0.7 [b]	9.08 ± 0.71	10.01±0.47 [a b]	8.45±0.37	n/s	p≤0.0001	p=0.01
**Hemoglobin (g/dL)**	15.6±1.1	14.95 ± 1.4	16.61±0.87 [a b]	14.62±0.53	n/s	p=0.002	n/s
**Mean corpuscular volume – MCV (fL)**	48.29±0.51 [b]	47.58 ± 0.29 [b]	48.13±0.29 [b]	49.67±1.4	p=0.003	n/s	p=0.001
**Mean corpuscular hemoglobin – MCH (pg)**	16.57±0.19 [b]	16.43 ± 0.32 [b]	16.6±0.15 [b]	17.22±0.59	p=0.004	n/s	p=0.007
**Mean corpuscular hemoglobin concentration – MCHC(g/dL)**	34.38±0.36	34.56 ± 0.44	34.47±0.22	34.67±0.54	n/s	n/s	n/s
**Hematocrit (%)**	45.36±3.26	43.22 ± 3.7	48.18±2.36 [a b]	42.18±1.19	n/s	p=0.001	n/s
**Fibrinogen (mg/dL)**	285.0 ± 37.4 [ab]	148.2 ± 48	308.3±52.69 [a b]	150.0±37.4	n/s	p≤0.0001	n/s
**Platelets (1000/mm³)**	1185±98.4 [a b]	803.33±188.24	1282.5±102.6 [a b]	954.5±115.4	p=0.043	p≤0.0001	n/s

Results expressed as the means ± standard deviations. n/s = not significant. [a] p > 0.05 compared to the PM group. [b] p > 0.05 compared to the LPS+PM group.

Table S3. Immunohistochemistry standards.

	Antibody	Concentration
**Neutrophils**	Anti – MPO (Abcam – cod. ab9535)	1:750
**Lymphocytes**	Anti - CD3 (Serotec – cod. MCA1477)	1:800 (Lung)<br>1:300 (Spleen)
**Macrophages**	Anti - MAC 2 (Cedarlane – cod. CL8942AP)	1:100000
**IL – 1ß**	Anti – IL-1ß (Santa Cruz – sc-7884)	1:400
**IL – 6**	Anti – IL-6 (Santa Cruz – sc-1265)	1:1500
**IL – 10**	Anti – IL-10 (Santa Cruz - sc-73309)	1:100
**TNF - α**	Anti – TNF –α (Santa Cruz –sc-1348)	1:3000
**MMP-2**	Anti – MMP2 (Santa Cruz – sc-8835)	1:500
